# Exposure to body odours combined with the effect of mindfulness treatment in patients with depressive and social anxiety symptoms - A preliminary study

**DOI:** 10.1192/j.eurpsy.2022.1899

**Published:** 2022-09-01

**Authors:** E. Vigna, V. Carli, G. Hadlaczky, D. Wasserman

**Affiliations:** 1 Karolinska Institutet, Lime - Department Of Learning, Informatics, Management And Ethics, Solna, Sweden; 2 Karolinska Institutet, Learning, Information, Management And Ethics, Stockholm, Sweden; 3 Karolinska Institutet, National Centre For Suicide Research And Prevention Of Mental Lll-health, Solna, Sweden

**Keywords:** Depression, Chemosignals, social anxiety, Body odour

## Abstract

**Introduction:**

To understand the way chemistry influences human communication is important since the reaction to chemosignals has many implications for science and society. For instance, previous research showed a connection between olfaction and affective psychiatric disorders. Olfactory processing may be impaired in subject presenting depression symptoms (DEP). Furthermore, a heightened sensitivity to social odours has been shown in subject with social anxiety symptoms (SAD). This may be due to the partial overlap of brain areas which are involved in olfactory processing and the pathophysiology of these disorders. Yet, more detailed research on the olfactory processing is required.

**Objectives:**

POTION is an EU funded project within the Horizon2020 initiative that aims to understand the nature of chemosignals in humans and their sphere of influence on social interaction. Within this project, we conducted a preliminary exploratory study examining whether the odours may be utilized to support positive outcomes of psychological therapy. It evaluates the catalyst effect of the odour conditions on the effectiveness of mindfulness meditation for SAD and DEP.

**Methods:**

Thirty subjects per patient group (total=60) are randomly allocated to one exposure group (happy or fearful human body odour or clean air) and follow the intervention while being exposed to the odour. Psychological outcome is measured before and after the intervention through the State-Trait Anxiety Inventory and the Profile of Mood State questionnaires. Analysis of variance is performed to assess outcome differences between groups.

**Results:**

Preliminary results on a subsample of 32 patients show a trend of deeper reduction of anxiety symptoms at post-treatment among odour-exposed groups compared to clean air (F(1,17)=11.08, p=0.004).

**Conclusions:**

Final results on the complete sample will be available and presented at the time of the congress.

**Disclosure:**

No significant relationships.

